# Central Corneal Thickness Measurements in Nonarteritic Anterior Ischemic Optic Neuropathy Patients: A Controlled Study

**DOI:** 10.1155/2014/904373

**Published:** 2014-04-02

**Authors:** Haneen Jabaly-Habib, Modi Naftali, George Habib

**Affiliations:** ^1^Ophthalmology Unit, Baruch Padeh Hospital, 15208 Poriya, Israel; ^2^Bar Ilan University, 1311502 Zfat, Israel; ^3^Department of Medicine, Carmel Medical Center, Haifa, Israel

## Abstract

*Purpose*. To measure central corneal thickness (CCT) in patients with history of nonarteritic anterior ischemic optic neuropathy (NAION). *Patients and Methods*. Patients older than 40 years with a history of NAION (group 1) were prospectively evaluated including full eye examination and central corneal thickness (CCT) pachymetry. Patients with a history of intraocular surgery, corneal disease, glaucoma, and contact lens wear were excluded. Measurements were also performed in a gender and age matched control group (group 2). *Results*. Thirty-one eyes of 31 NAION patients in group 1 were included and 30 eyes of 30 participants in group 2. There were 15 men in group 1 and 9 in group 2 (*P* = 0.141), and mean age of the patients was 59 ± 10 years in group 1 versus 61 ± 11 years in group 2 (*P* = 0.708). Mean CCT was 539 ± 30 microns in group 1 and 550 ± 33 microns in group 2 (*P* = 0.155). *Conclusion*. Patients with NAION have no special characteristic of CCT in contrast to the crowded optic disc known to be a significant anatomic risk factor for NAION. More studies should be carried out to investigate CCT and other structure related elements in NAION patients.

## 1. Introduction

Nonarteritic anterior ischemic optic neuropathy (NAION) is the most common optic neuropathy after the fifth decade of life. Its estimated incidence is 2.3–10.2 cases per 100,000 population per year [1]. It could be associated with moderate to severe visual loss. Risk factors include hypertension, diabetes mellitus, hypercholesterolemia, and crowded structure of the optic disc with small cup to disc ratio [2–5]. The later risk factor was first discovered by Beck et al., who believed that ischemia was the primary factor aggravated by the compressive effect at the lamina cribrosa level [3].

Central corneal thickness (CCT) was extensively studied in glaucoma but not in NAION patients. It has been proposed that the relation of CCT and glaucoma represents not just an artifact of applanation tonometry but also a biomechanical relation between CCT and the support structures of the optic nerve as stated by Brandt [6]. In the study of Morita et al., in which they studied corneal biomechanical properties in patients with normal tension glaucoma, they found that corneal resistance factor as well as corneal hysteresis were reduced in these patients which could lead to weakness of the lamina cribrosa ending up in glaucomatous damage of the optic nerve [7]. This issue is not solved yet. On the other hand, there are clues that suggest some relationship between corneal and optic disc structures such as corneal astigmatism in patients with tilted discs [8, 9]. Jonas et al., in two separate studies, found interestingly that CCT, horizontal, vertical corneal diameters, and anterior corneal curvature radius significantly and positively correlated with optic disc area. This suggested that eyes with small optic disc have small and thin cornea [10, 11]. CCT measurements in NAION patients, known to have crowded optic discs, were never reported in the English literature before. In this study we measured the CCT in NAION patients compared to a gender and age matched control group. This study hypothesis was that NAION patients might have thinner CCT that makes their crowded optic discs more prone to optic nerve damage by NAION.

## 2. Methods

Charts of all patients who were admitted due to swollen optic disc with visual loss, between the years 1999 and 2011, were reviewed. Patients diagnosed with NAION with at least one follow-up visit that confirmed the diagnosis were included in the study. 50 patients were eligible to the study and contacted by telephone. 31 patients agreed, were available for evaluation, and did not undergo intraocular surgery since their last examination. All patients underwent full eye examination and corneal thickness ultrasound pachymetry (group 1). Measurements were also performed in a gender and age matched group of patients who attended the outpatient clinic with no history of NAION (control group, group 2).

### 2.1. Inclusion Criteria

Age > 40 years and history of NAION diagnosed as acute visual loss associated with swollen disc and visual field defect, compatible with NAION, with at least one follow-up visit that confirmed the diagnosis.

### 2.2. Exclusion Criteria

History of intraocular surgery, corneal disease, glaucoma, or contact lens wear.

The CCT measurements were performed by ultrasound pachymeter (Corneogage plus-Sonogage, Cleveland, USA). Ten readings were performed for each eye; mean and standard deviation values were calculated.

The main outcome measure was the difference of CCT between the NAION and the control groups. To detect the difference of 30 microns between the two groups, at 5% level of significance and a power of 80%, a sample size of 29 participants in each group was needed.

For statistical analysis Chi square test and Student's *t*-test were used to compare categorical and continuous parameters, respectively, between the two groups.

The study was approved by the Helsinki ethics committee of Baroch Pade hospital, Poria.

## 3. Results

Thirty-one eyes of 31 NAION patients and 30 eyes of 30 control participants were included. Time elapsed from event was more than 12 months in the study group (mean 32.8 ± 15 months). There was no significant difference in age (*P* = 0.708) or gender distribution (*P* = 0.141) between group one and group two. There was no significant difference in CCT of the right and the left eye in both groups. The mean difference of CCT between right and left eye in group 1 and group 2 was 6 ± 5.5 microns and 7.7 ± 7.4 microns, respectively. The data of the study group is presented in Table 1. In the unilateral NAION patients the CCT of the NAION eye was included. In the subgroup with bilateral NAION (*n* = 7) the CCT of one eye was included due to the high resemblance of CCT between right eye (mean 516.6 ± 3.15 microns) and left eye (mean 523.8 ± 4.3 microns). Mean CCT was not statistically different between the NAION and the control groups (Table 2). In the subgroup with bilateral NAION only a tendency to have thinner cornea was found, compared to the control group.

## 4. Discussion

In this study central corneal thickness was not significantly different between NAION patients and the control groups. Since thin cornea seemed to be a risk factor for glaucoma as mentioned before and in CRVO contralateral eye [12], as well as the positive correlation found by Jonas et al. between disc diameter and corneal thickness [10, 11], the speculation of this study was to find thin cornea in NAION patients in addition to the crowded optic discs, a known anatomic risk factor to NAION [3]. This could be attributed to the small number of patients in this study, especially the subgroup with bilateral NAION (*n* = 7), who might have significantly thinner corneas. Recruiting such patients could be more complicated due to their visual limitations. This point will be considered in future studies, in our logistic arrangements.

There are several limitations in this study; no measurements of optic discs were performed, although in most of our NAION patients a crowded optic disc was seen clinically in the contralateral eye and documented. Although this preliminary study has negative results, still more “eyeball structure related” or anatomical studies are needed in NAION patients. For future studies corneal thickness, hysteresis, and ocular response analyser measurements in NAION patients can be measured. The biomechanical properties are being investigated in glaucoma patients [13], and it might be interesting to understand their implications in NAION patients as well. These measurements could help in extrapolating other risk factors for NAION and in which subgroups second eye is more prone to be involved.

## Figures and Tables

**Table 1:** Demographics among NAION group (*n* = 31).

Female	16
Male	15
Age	59+/−10
DM	14 (45%)
HTN	13 (41.9%)
Bilateral NAION	7 (22.5%)
Mean VA ± SD on admission of the affected eye	0.27 ± 0.29
Intraocular pressure ± SD	14.6 ± 2.22 mmHg
Crowded disc	18 (58%)

NAION: nonarteritic ischemic optic neuropathy; DM: diabetes mellitus; HTN: hypertension; VA: visual acuity; SD: standard deviation.

**Table 2:** Mean CCT in NAION and control groups.

	Group		
CCT	NAION group (*n* = 31)	Control group (*n* = 30)	*P* value*
CCT ± SD microns	539 ± 30	550 ± 33	*P* = 0.155

CCT: central corneal thickness, NAION: nonarteritic ischemic optic neuropathy, and SD: standard deviation.
